# Prognostic relevance of lymph node regression on survival in esophageal cancer: a systematic review and meta-analysis

**DOI:** 10.1093/dote/doab021

**Published:** 2021-04-24

**Authors:** Eliza Hagens, Karina Tukanova, Sara Jamel, Mark van Berge Henegouwen, George B Hanna, Suzanne Gisbertz, Sheraz R Markar

**Affiliations:** 1 Department of Surgery, Amsterdam UMC, University of Amsterdam, Cancer Center Amsterdam, Amsterdam, the Netherlands; 2 Department of Surgery and Cancer, Imperial College London, London, UK

**Keywords:** esophageal cancer, lymph node regression, neoadjuvant therapy

## Abstract

**Introduction:**

The prognostic value of histomorphologic regression in primary esophageal cancer has been previously established, however the impact of lymph node (LN) response on survival still remains unclear. The aim of this review was to assess the prognostic significance of LN regression or downstaging following neoadjuvant therapy for esophageal cancer.

**Methods:**

An electronic search was performed to identify articles evaluating LN regression or downstaging after neoadjuvant therapy. Random effects meta-analyses were performed to assess the influence of regression in the LNs and nodal downstaging on overall survival. Histomorphologic tumor regression in LNs was defined by the absence of viable cells or degree of fibrosis on histopathologic examination. Downstaged LNs were defined as pN0 nodes by the tumor, node, and metastasis classification, which were positive prior to treatment neoadjuvant.

**Results:**

Eight articles were included, three of which assessed tumor regression (number of patients = 292) and five assessed downstaging (number of patients = 1368). Complete tumor regression (average rate of 29.1%) in the LNs was associated with improved survival, although not statistically significant (hazard ratio [HR] = 0.52, 95% confidence interval [CI] = 0.26–1.06; *P* = 0.17). LNs downstaging (average rate of 32.2%) was associated with improved survival compared to node positivity after neoadjuvant treatment (HR = 0.41, 95%CI = 0.22–0.77; *P* = 0.005).

**Discussion:**

The findings of this meta-analysis have shown a survival benefit in patients with LN downstaging and are suggestive for considering LN downstaging to ypN0 as an additional prognostic marker in staging and in the comparative evaluation of differing neoadjuvant regimens in clinical trials. No statistically significant effect of histopathologic regression in the LNs on long-term survival was seen.

## INTRODUCTION

The incidence of esophageal cancer is rapidly increasing, affecting > 450 000 people worldwide.^1^ Surgical resection is the mainstay of curative treatment of resectable esophageal cancer.^2^ Nevertheless, the 5-year survival rate of patients treated with surgery alone is only 15–24% due to the high incidence of locally advanced disease and distant metastases.^3–5^ As a consequence, several studies have investigated the benefits of neoadjuvant regimens, which aim at downstaging the primary tumor and reducing micrometastatic disease. An improved survival of neoadjuvant chemotherapy or chemoradiotherapy over surgery alone was found whilst preoperative radiotherapy alone failed to increase survival.^6–10^

Histomorphologic regression is defined as regressive changes based on histopathologic evaluation and is commonly reported by means of the Mandard and Becker grading systems. In the primary tumor, the tumor regression grade has demonstrated its use in the prognostic assessment.^11^^,^^12^ In addition, the nodal status determined by the tumor, node, and metastasis (TNM) classification prior to and following chemoradiotherapy seems to equally affect the survival. Patients without lymph node metastases have better overall survival regardless the tumor regression grade.^13–16^ Controversial results were published by a randomized controlled trial comparing the prognostic impact of nodal response in surgery alone compared to neoadjuvant chemoradiotherapy followed by surgery.^17^ Patients with persistent lymph node positivity following neoadjuvant treatment showed worse outcome compared to patients with positive nodes treated by surgery alone. To date, the prognostic value of nodal response remains unclear.

The primary aim of this review is to evaluate the prognostic relevance of lymph node (LN) regression following neoadjuvant chemotherapy or chemoradiotherapy in patients treated for esophageal adenocarcinoma (AC) or squamous cell carcinoma (SCC) and to assess the prognostic relevance of LN downstaging in these patients. The secondary aim was to assess the relationship between primary tumor and nodal response.

## METHODS

This systematic review was performed in line with Cochrane recommendations, following the meta-analysis of observational studies in epidemiology guidelines and preferred reporting items for systematic reviews and meta-analyse (PRISMA) statement.^18^^,^^19^

### Search Strategy and Study Selection

A systematic literature search was performed of the Cochrane Library, MEDLINE and EMBASE databases on 20th September 2019. The following terms were used (including synonyms and closely related words) as index terms or free-text words: ‘(o)esophageal cancer (both esophageal AC and SCC)’, ‘lymph node metastases’, and ‘regression’. The full search strategies for PubMed and Embase.com can be found in Appendix 1. In addition, the reference lists of included articles were searched to further identify relevant studies.

Articles were independently evaluated by three reviewers (EH, KT, and SJ) in two stages: screening of titles and abstracts followed by the retrieval and screening of full-text articles. Publications were included in this review if they met each of the following criteria:

1. An esophageal resection with two- or three-field lymphadenectomy with curative intent was performed in patients with esophageal AC or SCC.
2. Either chemotherapy or chemoradiotherapy was administered as neoadjuvant therapy.
3. Comparative studies of patients which compared no response in the lymph nodes to:
  - Regression: complete or partial tumor regression in the lymph nodes;or
  - Downstaging: pathologic complete response ypN0.

Publications assessing tumor regression in the lymph nodes were excluded in case of non-comparative studies, or comparative studies failing to make a clear division between patients with complete or subtotal regression and partial or no regression in the lymph nodes regarding survival outcome. Publications were also excluded if the study population did not receive neoadjuvant treatment, or in case of case reports, review articles, poster abstracts, or animal studies.

### Data extraction

The following data were extracted from each study: first author, year of publication, study design, sample size, histologic subtype, clinical and pathologic T- and N-staging, staging tool, neoadjuvant treatment modality, surgical approach, and extent of lymphadenectomy and are described Tables 1 and 2. For the articles assessing LN regression, the classification of regression was also extracted. Hazard ratio’s (HRs) were extracted from the text or calculated from the Kaplan–Meier survival estimates if they were not provided as previously described by Markar *et al*.^20^

**Table 1:** Characteristics of included studies

Author	Year	Type of study	*N*	Staging method	Classification of lymph node response groups	
Regression					Complete/subtotal response	Partial/no response
Bollschweiler^22^	2011	Retrospective cohort study	40	Barium swallow, endoscopy, EUS, and CT chest and abdomen	LNMG: no LNM and < 3 LN with central fibrosis Medium risk LNMG: no LNM and 3 or more LN with central fibrosis or LN ratio < 5 (= number LN involved/number resected LN)	High risk LNMG: everything else
Nieman^23^	2015	Retrospective cohort study	69	CT or PET, and flexible upper endoscopy	Treatment response nodes with evidence of prior cancer involvement (acellular mucin pools, central fibrosis, necrosis or calcification) but no currently viable cancer cells	Positive nodes involved with malignancy.
Davies^24^	2018	Retrospective cohort study	183	CT, EUS, endoscopy, and fluorodeoxyglucose PET	Lymph node regression score created according to the proportion of fibrosis and residual tumor within the lymph node:	
					Score 1: complete response Score 2: <10 per cent remaining tumor	Score 3: 10–50 per cent remaining tumor Score 4: >50 per cent viable tumor Score 5: no response
**Downstaging**						
Rice^26^	2001	Retrospective cohort study	77	EUS and CT	TNM-classification: downstaged from cN1 to ypN0	
Donohoe^28^	2013	Retrospective cohort study	155	EUS and fluorodeoxyglucose PET/CT	TNM-classification: downstaged from cN1 to ypN0	
Zanoni^25^	2016	Retrospective cohort study	55	CT, EUS, endoscopy, and PET/CT	TNM-classification: downstaged from cN1 to ypN0	
Shapiro^15^	2017	Retrospective cohort study	100	CT, EUS, endoscopy, and PET/CT	TNM-classification: downstaged from cN1 to ypN0	
Noble^27^	2017	Retrospective cohort study	981	CT, EUS, and fluorodeoxyglucose PET/CT. Additional laparoscopy when indicated.	TNM-classification: downstaged from cN1 to ypN0	

*Abbreviations:* EUS, endoscopic ultrasound; LNM, lymph node metastases; LNMR, low risk lymph node morphologic grading; LNMRG, high response lymph node morphologic grading; TNM, tumor, node, metastasis (TNM) staging system by AJCC.

**Table 2:** Characteristics of study population

Author	Histology	cT-stage	(y)pT-stage	cN-stage	(y)pN-stage	Surgical approach	Extend of lymphadenectomy	Median LN yield (IQR)	Type of neoadjuvant treatment	
Regression									Chemotherapy	CRT
Bollschweiler^22^	AC (20) SCC (20)	cT3/4	NS	cN+	ypN0 23 ypN1 17	TTE	2-field lymphadenectomy	31 (24.5–38.5	/	40
Nieman^23^	AC (69)	cTx	NS	cN+	ypN0 18 ypN+ 51	TTE THE MIE	NS	NS	70	NS
Davies^24^	AC (183)	cT2–4	ypT0 3 ypT1/2 64 ypT3/4 116	cN+	ypN0 19 ypN1/3 164	NS	NS	NS	183	/
		**Downstaging**								
Rice^26^	AC (NS) SCC (NS) AC/SCC (NS)	cTis/1/2 14 cT3/4 55	ypT0 37 ypT3/4 32	cN1 69	ypN0 37 ypN1 40	TTE 58 THE 1	2-field lymphadenectomy; LN sampling	NS	/	69
Donohoe^28^	AC (NS) SCC (NS)	cTx	NS	cN1 130	ypN0 56 ypN1 99	TTE THE	NS	NS	/	130
Zanoni^25^	AC (25) SCC (15)	cT2 1 cT2/3 24 cT3 25 cT3/4 4 cT4 1	ypT0 29 ypT+ 26	cN1 55	ypN0 37 ypN+ 18	TTE	D1 and 2-field lymphadenectomy	ypN0 20 (15–29) ypN+ 22.5 (18–33)	/	130
Shapiro	AC (138) SCC (41)	cT1 7 cT2 38 cT3 135	ypT0 59 ypT1 32 ypT2 29 ypT3 60	cN0 57 cN1 74 cN2 46 cN3 3	ypN0 123 ypN1 40 ypN2 13 ypN3 4	TTE THE	2-field lymphadenectomy	NS	/	180
Noble^27^	AC (981)	cTx	NS	cNx	ypN0 259 ypN+ 722	NS	NS	NS	/	130

*Abbreviations:* AC, esophageal adenocarcinoma; CRT, chemoradiotherapy; LN, lymph nodes; MIE, minimally invasive esophagectomy; NS, not specified; SCC, esophageal squamous cell carcinoma; THE, transhiatal esophagectomy; TTE, transthoracic esophagectomy*.*

### Outcome measures and statistical analysis

The primary aim was to assess the prognostic impact of nodal status, both with pathological lymph node (y)pN data as well as with histomorphologic regression data.

The LN response was defined as regression from histomorphologic assessment or as downstaging using the TNM classification. For LN regression, a comparison was made between survival outcomes in patients with complete or subtotal tumor-regressed lymph nodes and those with only a partial or no regression in the lymph nodes. Included studies used different grading systems for histomorphologic regression in the lymph nodes.

To enable pooled analysis, complete or subtotal regression was defined as either by the absence of metastatic lymph nodes with evidence of prior cancer involvement (presence of central fibrosis, acellular mucin pools, necrosis, or calcification); absence of metastatic lymph nodes or lymph nodes ratio < 0.05 (number of involved lymph nodes divided by the number of resected lymph nodes); or < 10% of remaining tumor in the lymph nodes.

The remaining cases were considered as either partial or no LN regression.

Lymph nodes were considered as downstaged in patients initially staged as clinical positive lymph nodes, cN+ and became ypN0 following neoadjuvant treatment. This group was compared to patients with either cN+ypN+ or cN0ypN+ disease.

The secondary aim was to assess the relationship between primary tumor response and regression or downstaging in the lymph node.

The logarithm of the HR with 95% confidence intervals (CIs) was used as the primary summary statistic. The HR and its variance were estimated, either by extracting this directly from the study or by additional calculation depending on the method of data being presented: annual mortality rates, survival curves, or number of deaths.^21^ Meta-analyses of the data were performed using a random effects model. Heterogeneity amongst studies was assessed by means of the Cochran’s Q statistic, which is a null hypothesis in which *P* < 0.05 is taken to indicate the presence of significant heterogeneity. Furthermore, the *I**^2^*-inconsistency test was performed to measure the degree of variation not attributable to chance alone, which was graded as low (*I^2^* < 25%), moderate (*I^2^* = 25–75%), or high (*I^2^* > 75%). Statistical analyses were performed with the software Review Manager version 5.3.

## RESULTS

### Study characteristics

**Fig. 1:**
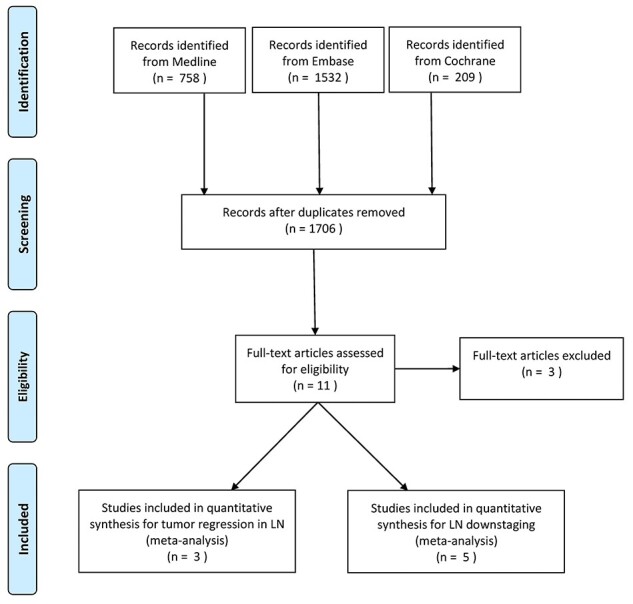
PRISMA flow chart of the study selection process. (LN, lymph node).

The systematic search yielded 2499 results. After removal of duplicates, 1706 references were screened on title and abstract. Subsequent screening of full text identified eight publications, three of which assessed tumor regression and five assessed downstaging.^22–29^ A graphical representation of the selection procedure is shown in a PRISMA flow chart (Fig. 1). Baseline characteristics of included studies and the study population, tumor type, diagnostic work-up, treatment approach, and classification of LN response are shown in Tables 1 and 2.

### Lymph node regression and overall survival

**Fig. 2:**
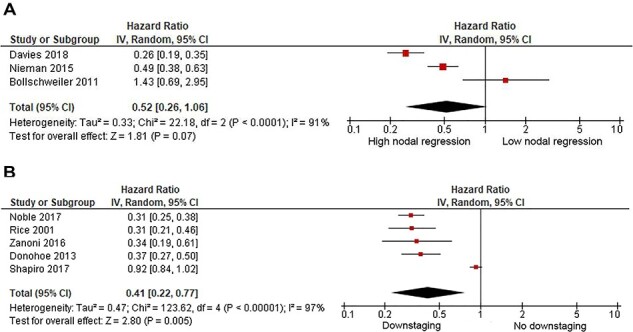
(a) Meta-analysis for influence of complete or subtotal versus partial or no regression on overall survival (partial or no regression as reference). (b) Meta-analysis on the influence of downstaging on overall survival (no downstaging as reference).

Pooled analysis of three publications included 292 patients, 106 of which had complete or subtotal regressed lymph nodes whilst 186 had a partial or no regression in the lymph nodes following treatment. This pooled data demonstrated that complete regression (average rate of 29.1%) in the lymph nodes was associated with improved survival, although this failed to reach statistical significance (HR = 0.52, 95% CI = 0.26–1.06; *P* = 0.07), Figure 2a. There was evidence of high statistical heterogeneity for this result (*I^2^* = 91%).

### Lymph node downstaging to ypN0 and overall survival

Pooled analysis of five publications included 1368 patients, of which 432 patients were downstaged. This pooled analysis showed that downstaging (average rate of 32.2%) in the lymph nodes had improved survival compared to ypN+ disease (HR = 0.41, 95% CI = 0.22–0.77; *P* = 0.005), Figure 2b. There was evidence of high statistical heterogeneity for this result (*I^2^* = 98%).

### Primary tumor response and lymph node regression

One study^22^ reported primary tumor regression by using the Cologne Regression scale,^15^^,^^30^ classifying patients with < 10% vital residual tumor cells as major response and the remaining cases as minor response. One paper^24^ used the scoring system as described by Mandard *et al*.^31^ and the last study^23^ reported the histologic grading only. In the study conducted by Bollschweiler *et al*.,^22^ the majority (65%) of the patients with a minor response in the primary tumor presented with LN metastasis, whilst this was the case for only 20% of the patients with a major response (*P* = 0.01). In the study by Davies *et al*.,^24^ primary tumor regression was classified as a Mandard score of 1–3. The authors however, defined LN response as score 1, complete regression, to score 3, with 10–50% viable tumor cells present. A similar proportion of patients with and those without LN response were found in case of a Mandard score of 1–3 in the primary tumor (56 and 44%, respectively). In contrast, 27% of the patients with a Mandard score of 4 or 5 had a LN regression score of 1–3.

### Primary tumor response and lymph node response according to TNM-classification

Three out of five included papers assessed primary tumor response alongside LN downstaging to ypN0. In the study by Donohoe *et al*.,^28^ the complete primary tumor regression group had the lowest proportion of patients with ypN+ disease (7%). Nodal involvement increased with decreasing response in the primary tumor following treatment, with 51 and 84% having ypN+ disease in patients with partial and no response in the primary tumor, respectively (*P* < 0.0001). Zanoni *et al*.^25^ assessed overall survival for LN downstaging and disease-free survival for combined pathologic primary tumor and LN status. Patients with both downstaged lymph nodes to ypN0 and primary tumor had a 3-year disease-free survival of 80% whilst this dropped to 23% in case of downstaged lymph nodes with presence of residual tumor at the primary site (*P* = 0.003). Similarly, the majority (59.9%) of patients with a complete or subtotal primary tumor regression experienced a downstaging in the lymph nodes compared to 23.3% in case of a partial or no regression in the primary tumor (*P* < 0.001) in the study by Noble *et al*.^27^

## DISCUSSION

This systematic review and meta-analysis summarizes the existing evidence regarding the prognostic relevance of LN regression and downstaging in patients who have undergone neoadjuvant treatment and surgical resection for esophageal cancer. A prognostic benefit was seen in patients with LN response following neoadjuvant treatment, in patients with complete or subtotal regression within the lymph nodes as well as in case of nodal downstaging. Furthermore, a discrepancy between the lymph nodes and primary tumor response was noted, further supporting the importance of considering nodal response as an independent prognostic factor in addition to primary tumor response.

Several studies suggested that complete response of the primary esophageal cancer, both pathologic and histomorphologic, was associated with improved survival.^17^^,^^32–36^ In the literature, different grading systems are used to assess the histomorphologic regression of the primary tumor, such as proposed by Mandard *et al*.^31^ and classification according to the Cologne Regression Scale.^15^^,^^30^ In contrast, no standardized scales have been established yet for histomorphologic LN regression, which remains an important area for standardization.

Furthermore, the impact of histomorphologic LN regression on survival previously was unclear and incongruous results published regarding the association between primary tumor and LN responses. Although primary tumor regression and pathologic LN status seemed to be independent prognostic factors,^30^^,^^37^ a significant association has been found between these responses, showing improved survival in patients with complete or subtotal primary tumor regression and the absence of LN metastasis.^30^^,^^38^ In contrast with these findings, Makino *et al*. demonstrated that although primary tumor and LN responses on 18-fluorodeoxyglucose-positron emission tomography were significant and equal in magnitude following neoadjuvant treatment, there was no significant correlation between them.^39^^,^^40^ The latter results were supported by a recent study (Urakawa *et al*., 2019), establishing a weak correlation between primary tumor and LN response, defined as area reduction of at least 60% and size reduction of at least 30%, respectively. Moreover, these authors argued that primary tumor response was significantly associated with local recurrences, whilst LN response was an independent prognostic factor for disease-free survival following neoadjuvant chemotherapy.^41^

In this meta-analysis, two out of three included articles showed improved survival following complete or subtotal histomorphologic LN regression. Meanwhile, Bollschweiler *et al*.^22^ presented a contrasting result, showing better survival outcome in the partial/no regression group. This finding might however be explained by the small size in this study. Furthermore, two articles also assessed the histomorphologic primary tumor response alongside LN regression. One study^22^ found a significant correlation between primary tumor and LN regression, while the other study^24^ showed that the proportion of patients with and without LN response were similar in the group experiencing a primary tumor response. In this study regression scores 1–3 were however assessed together for both primary tumor and LN regression, thus not separating complete from partial regression. Nevertheless, there was a considerable proportion of patients (27%) in this study with a LN regression score of 1–3 in case of a primary tumor without response to neoadjuvant treatment (Mandard score 4 or 5). This finding highlights the discrepancy between the LN and primary tumor response, further supporting the importance of considering nodal response as a prognostic factor in addition to primary tumor response.

Similarly, three of the five studies assessed the primary tumor response alongside LN downstaging and found a greater proportion of patients with downstaged LN in the group of complete or subtotal primary tumor regression. These findings were consistent with the results published by Reynolds *et al*.,^38^ establishing a significant association between complete regression in the primary tumor and downstaged LN (*P* < 0.05).

In the series by Reynolds *et al*., only chemoradiotherapy was used as neoadjuvant treatment in articles assessing pathologic LN downstaging. Meanwhile, the use of neoadjuvant treatment modalities varied across studies assessing regression in the present study, as patients were treated with either chemotherapy or chemoradiotherapy. Nevertheless, a recent randomized trial found no significant difference in survival comparing both regimens in esophageal or gastro-esophageal junction cancer.^42^^,^^43^

Important limitations were present in this systematic review and meta-analysis, which must be considered in the interpreting the results. First, all of the included studies had a retrospective, observational design, increasing the risk for selection bias. To date, no standardized LN regression grading system has been described. Therefore, histomorphologic LN regression was defined differently by the authors in every study included in the analysis. Classification of LN regression was however consistent and clearly defined in this meta-analysis. Complete LN regression was characterized by the absence of metastasis and with evidence of prior cancer involvement (central fibrosis, acellular mucin pools, necrosis, or calcification), or a lymph nodes ratio of <0.05. This patient group and those with subtotal regression, defined as LN with <10% of remaining tumor, were analyzed as one group. The remaining cases were considered as partial or no regression in the LN. This approach is similar to the classification described by Junker *et al*.,^44^ comparing complete or subtotal (<10% of remaining tumor) regression to partial (10–50% residual tumor) or no regression (>50% residual tumor) of non-small-cell lung cancer following neoadjuvant chemoradiation. Similarly, Schneider *et al*.^30^ divided the former regression grades into minor and major response, consisting of complete or subtotal regression, and partial or no regression, respectively, since no significant difference was found in median survival within these groups. Moreover, other authors divide patients in groups with complete, partial or no response by combing patients with sequent Mandard grades.^27^^,^^28^^,^^45^ As a result, papers were excluded from the current meta-analysis in which patients with complete or subtotal regression and those with a partial response were not separated on histomorphology as described in our classification. Furthermore, a meta-analysis by Visser *et al*. assessed the relationship between LN yield and survival. Due to variation in defining the threshold for low and high yield, the authors have compared the lowest and highest LN yield groups for each study, showing significantly increase in overall and disease-free survival in case of a high LN yield (*P* < 0.01).^46^ In our meta-analysis, patient demographics and study characteristics, including LN yield could not be extracted for all studies since patients undergoing neoadjuvant therapy followed by surgical excision for whom LN response was assessed, were included in the meta-analysis. Also, some of the included studies included patients which were operated transhiatally and thus no adequate resection of the lymph nodes could be guaranteed, subsequently this lowers the reliability to assess LN response. In addition, both meta-analyses showed to have a high *I^2^*, which indicates considerable heterogeneity. However, *I^2^* can be biased in a small meta-analysis and thus might be unreliable in the present study.^47^ Furthermore, no subgroup analysis was performed for the histological subtypes. In this meta-analysis, only the proportion of participants was included for each study, who had undergone neoadjuvant treatment followed by surgery. Two out of eight included studies provided the histological subtype for the total sample size only, thus not indicating the histological subtype specifically for the patients who have undergone neoadjuvant followed by esophagectomy. Previously, higher locoregional and distance recurrence rates have been reported by Mariette *et al*. in patients with AC.^48^ However, overall recurrence rates, postoperative mortality and morbidity were similar for both AC and SCC in the same study (*P* > 0.084, *P* = 0.078, and *P* = 0.077, respectively). Lastly, there is evidence that cN staging is inaccurate.^49^ The majority of included studies used computed tomography (CT), positron emission tomography (PET) and endoscopic ultrasounds (EUS) for assessment of clinical nodal status. Results from three meta-analyses found a pooled sensitivity and specificity of EUS ranging between 76–84% and 70–85%, respectively.^50–52^ Furthermore, Sgourakis *et al*. also reported sensitivity and specificity of 59 and 81% respectively for Fluorodeoxyglucose- PET, compared to 52 and 80% for CT-staging.^51^ Therefore classification of down staging from cN+ nodes to ypN0 might also be inaccurate in some of the included studies. This may have confounded the results in the present meta-analysis.

In conclusion, the findings of this meta-analysis have shown a survival benefit in patients with LN downstaging and are suggestive for considering LN downstaging to ypN0 as additional prognostic markers in staging and in the comparative evaluation of differing neoadjuvant regimens in clinical trials. Tumor response in LNs seems important but there is a current lack of quality data to really show to what extent. Future investigations should investigate how ypN0 can accurately be identified and whether down staging to ypN0 allows for a more patient-tailored surgical approach.

## FUNDING

Mr Sheraz Markar is funded by the National Institute of Health Research (NIHR). The views expressed are those of the authors and not necessarily those of the National Health Service (NHS), the NIHR, or the Department of Health.

## CONFLICTS OF INTERESTS

The authors declared no conflict of interest.

## APPENDIX I

**Table:** Search strategy

**Database**	**Search**	**Query**	**Items found**
**Cochrane**	#1	((((Adenocarcino^*^ OR squamous OR SCC OR malign^*^ OR tumour OR cancer OR neoplasm^*^):ti,ab,kw) OR (MeSH descriptor: [Adenocarcinoma] explode all trees) OR (MeSH descriptor: [Carcinoma, Squamous Cell] explode all trees) OR (MeSH descriptor: [Neoplasms, Squamous Cell] explode all trees)) AND ((esophag^*^):ti,ab,kw))) OR (((esophagectomy):ti,ab,kw) OR (MeSH descriptor: [Esophagectomy] explode all trees) OR (MeSH descriptor: [Esophageal Neoplasms] explode all trees))	6053	
	#2	(((lymph node^*^ OR nodal metastas^*^):ti,ab,kw) OR ((lymph^*^ near/2 metastas^*^):ti,ab,kw) OR (MeSH descriptor: [Lymph Nodes] explode all trees) OR (MeSH descriptor: [Lymphatic Metastasis] explode all trees))AND (((((Regression OR remission):ti,ab,kw) OR (MeSH descriptor: [Remission Induction] explode all trees)) OR (lymph node response):ti,ab,kw))	3108	
	#3	#1 AND #2	209	
**Embase**	#1	((Adenocarcino^*^.mp. OR squamous.mp. OR SCC.mp. OR tumor.mp. OR tumour.mp. OR cancer.mp. OR neoplasm^*^.mp. OR malign^*^.mp. OR exp ADENOCARCINOMA/OR exp CARCINOMA, SQUAMOUS CELL/or exp NEOPLASMS, SQUAMOUS CELL/) AND (esophag^*^.mp. OR oesophag^*^.mp.)) OR (esophagectomy.mp. OR oesophagectomy.mp. OR exp ESOPHAGECTOMY/OR exp Esophageal Neoplasms/)	102 737	
	#2	(regression.mp. OR remission.mp. OR exp Remission Induction/) AND ((lymph^*^ adj2 metastas^*^).mp. OR lymph node^*^.mp. OR nodal metastas^*^.mp. OR exp Lymphatic Metastasis/OR exp Lymph Nodes/) OR (lymph node response.mp.)	33 210	
	#3	#1 AND #2	1532	
**Medline**	#1	((Adenocarcino^*^.mp. OR squamous.mp. OR SCC.mp. OR tumor.mp. OR tumour.mp. OR cancer.mp. OR neoplasm^*^.mp. OR malign^*^.mp. OR exp ADENOCARCINOMA/OR exp CARCINOMA, SQUAMOUS CELL/or exp NEOPLASMS, SQUAMOUS CELL/) AND (esophag^*^.mp. OR oesophag^*^.mp.)) OR (esophagectomy.mp. OR oesophagectomy.mp. OR exp ESOPHAGECTOMY/OR exp Esophageal Neoplasms/)	63 015	
	#2	(regression.mp. OR remission.mp. OR exp Remission Induction/) AND ((lymph^*^ adj2 metastas^*^).mp. OR lymph node^*^.mp. OR nodal metastas^*^.mp. OR exp Lymphatic Metastasis/OR exp Lymph Nodes/) OR (lymph node response.mp.)	16 105	
	#3	#1 AND #2	758	

MeSH, medical subject headings; Ti, ab, kw, words in title OR abstract OR keyword; mp = keyword; Exp/ = exploded MeSH term.
